# An exploration of self-management support in the context of palliative nursing: a modified concept analysis

**DOI:** 10.1186/1472-6955-13-21

**Published:** 2014-07-23

**Authors:** Bridget Johnston, Liz Rogerson, Jurate Macijauskiene, Aurelija Blaževičienė, Patricia Cholewka

**Affiliations:** 1 Sue Ryder Care Centre for the Study of Supportive, Palliative and End of Life Care, School of Health Sciences, University of Nottingham, Queen’s Medical Centre, Derby Road, Nottingham NG7 2HA, UK; 2 School of Nursing and Midwifery, University of Dundee, 11 Airlie Place, Dundee Scotland, DD1 4HJ, UK; 3 Faculty of Nursing, Lithuanian University of Health Sciences, Mickeviciaus 9, Kaunas, LT-44307, Lithuania; 4 Department of Nursing, New York City College of Technology, CUNY, 300 Jay Street, P-505, Brooklyn, NY 11201, USA

**Keywords:** Self-care, Self-management, Palliative, Nursing, Death and dying, Education, Concept analysis, Literature review

## Abstract

**Background:**

The role of self-management is often ambiguous, yet, it is an important area in clinical practice for palliative nurses. A clear conceptual understanding, however, of what it represents is lacking.

**Method:**

This paper reports an analysis of the concept of self-management support in palliative nursing.

Avant and Walker’s method was used to guide this concept analysis.

A search of electronic databases (1990–2013), use of internet search engines and supplementary hand searching produced an international data set of reviews, empirical research, editorials, protocols and guidelines.

**Results:**

Based on the analysis self-management support in palliative nursing has been defined as assessing, planning, and implementing appropriate care to enable the patient to live until they die and supporting the patient to be given the means to master or deal with their illness or their effects of their illness themselves.

**Conclusions:**

Clarity with the concept of self-management support and palliative nursing could enable nurses to provide more patient and family centred care to people facing life threatening illnesses.

## Background

The concept of self-management is not new and can be traced back in the UK to the start of the 20^th^ century [1]. Nevertheless, despite its longevity, defining self-management as a concept and clarifying its meaning for, and use, in practice has been found to be particularly challenging in the field of palliative care. Major obstacles to seeking clarity on self-management as a concept are that in spite of a plethora of available literature on self-management, the dominant focus is on managing chronic disease. However managing chronic illness presents patients and professionals with very different challenges to those found in palliative care [2]. An example of this is a UK government document, which defines self-care as ‘the actions people take for themselves, their children and their families to stay fit and maintain good physical and mental health; meet social and psychological needs; prevent illness or accidents; care for minor ailments and long term conditions; and maintain health and wellbeing after an acute illness or discharge from hospital’ [3]. Issues which are not the main focus in advanced disease when people are seriously ill, may lack functional capacity, and may be dependent on others for help.

These issues and challenges are compounded by the ambiguity found in the terminology surrounding self-management, including the interchangeable use of similar terms such as self-care, self-efficacy, self-help [2,4]. In a thematic analysis of the conceptualisation of self-care, self-management and self-management support in the long term conditions management literature, Jones et al. [5] found that the terms differed regarding the nature of the *imperative for action*. For instance, self-care is inevitable but includes choice; self-management is an inevitable activity whereas self-management support is an essential activity. Self-management support lies within context of coordinated networks derived from the health and social care system. This process is person-centred and any imperative for action is derived from the collaboration between people with long term conditions and thus improving support services. This concept analysis, therefore, is particularly focussed on self-management support.

Political interest in self-management has also increased over the last decade, internationally, as governments attempt to contain the costs of health care, alongside addressing goals to improve health outcomes, patient satisfaction ratings and service delivery [3,6]. This shift in the balance of care addresses the challenges associated with the increasing number of people suffering long-term conditions. Professionals, patients and politicians alike are, therefore, part of a trend in seeking health care and health service delivery solutions that are more patient focused and recognise the central role of patients in the care process.

A factor that can affect self-care behaviors is health literacy. Health literacy is “cognitive and social skills which determine the motivation and ability of individuals to gain access to, understand and use information in ways which promote and maintain good health” [7]. Health Literacy includes skills and behaviours, that all people need, to for instance, find their way to the right place in a hospital, to fill out medical forms, and to communicate with healthcare providers. Poor literacy and numeracy skills can, therefore, result in difficulties in interpreting and performing self-care activities.

This concept analysis was conducted as part of a larger study: Integrating Self-Management and Palliation Concepts (IMPACT), which was commissioned and funded by the ATLANTIS programme (Actions for Transatlantic Links and Academic Networks in Training and Integrated Studies) aimed at facilitating cooperation in higher education and vocational training in the European Union and the United States. The original study involved a partnership of four universities in Scotland, Lithuania, New York and Ohio. Two aims of IMPACT were the development of a comparative framework for policy analysis pertaining to palliative care and self-management and the creation of policy benchmarks for the delivery of palliative care to guide best practice in the management of self-care practices, in palliative care nursing in Europe and the Unites States of America (USA).

### Why exploring self-management support and palliative nursing is important

Living with a life limiting condition, be it advanced cancer, or any other non-malignant disease, for which there is no cure, can have a devastating effect on a person. The impact can extend to psychological, social, physical, economic and cultural aspects of people’s lives [8-10]. Individuals tend to cope as well as they can, with the support they have, whether that be from family or other sources, but often they do not have the information, skills or knowledge to make well informed decisions or the appropriate response [11]. Many patients do not understand what health professionals have said to them and do not, therefore, participate in decisions about their care, this leaves them ill-prepared to make daily decisions and take actions that lead to good care management. A collaborative relationship between nurses, health care teams, and patients and their families is, therefore important. Supported self-management in palliative care, by nurses, can, therefore, empower people to acknowledge the impact of their condition on their life, and enable them, where possible, to face the range of challenges they may have, and identify areas where they need further support, help or care [8].

In seeking to address the above issues this paper is concerned with exploring the issue of how self-management support in palliative nursing is conceptualised in the literature.

## Methods

### Concept analysis

Concept analysis is considered a relatively new research approach, method or process [12] and is not an approach which is universally accepted [13]. The term concept analysis refers to the unfolding, exploring and understanding of concepts for the purposes of concept development, delineation, clarification, correction, identification, refinement and validation [14-17].

Walker and Avant [16] in the method chosen here, propose that; concepts are mental representations of a phenomenon or an idea, of an action or a thing that can accurately represents these occurrences within clinical practice. They advocate that the conceptualisation of concepts and their use in describing nursing practice is a stepping-stone towards the standardization of nursing language. In addition, concepts can be described as efforts to categorize information into meaningful constructs when applied to a phenomenon that occurs within the field of health care. A concept analysis is, therefore, a rigorous and precise process of operationalizing the defining characteristics and attributes of a phenomenon into a communicable understanding, and is undertaken using a structured framework [16].

Concept analysis is a method of conceptual knowledge representation and data analysis that can be used to clarify meanings and develop operational definitions, through considering evidence from multiple disciplines and sources. By applying a recognised methodological framework a more objective approach to concept clarification is accomplished. The systematic framework also means that the process is applicable within diverse scientific disciplines [16].

To guide the process of literature and analysis a modified version of the eight-step model presented by Walker and Avant was applied [16]. The eight steps of the model do not need to be, and were not, used in chronological order and can and were modified as the enquiry progressed.

Table 1 outlines the 8 steps and whether and how applied for this concept analysis.

**Table 1:** Walker and Avant concept analysis steps

**Step**	**Used in this concept analysis**
1. Select the concept	Yes (dictionary definition-methods)
2. Determining the aim or purpose of the analysis	Yes (research question and aim-methods)
3. Identifying all the known uses of the concept	Yes (literature review-methods and results)
4. Determining the defining attributes	Yes (literature review- results)
5. Identifying a model case (“real life” example, which contains all of the critical attributes	No
6. Identifying any of the borderline, related, contrary, invented and illegitimate cases	No
7. Identifying antecedents and consequences	Yes (literature review- results)
8. Identifying empirical referents	

### Selecting the concept

The concept self-management support and how it relates to palliative nursing will be analysed in this paper.

In Walker and Avant’s method the first stage is usually a literature review.

#### Search strategy

Primary data was identified by searching three online electronic databases via EBSCO Host: Medline, CinAHL and PsycINFO which cover literature from disciplines such as medicine, nursing, allied health, sociology and psychology. The key search terms are presented in Table 2.

**Table 2:** Search terms and key words

**Search string/number**	**Keywords**
1	Self care
2	Self management
3	Self management support
4	1 or 2 or 3
5	Palliative care
6	Terminally ill
7	Terminal care
8	Hospice
9	Life limiting illness
10	End of life care
11	Nurs$
12	5 or 6 or 7 or 8 or 9 or 10
13	4 and 11

Search string in Medline, CinAHL and PsycINFO, keywords: 1. self care.

The search led to 205 potential articles, with duplicates removed this left 165 to review. Of these 165 12 provided definitions of self-care and palliative care but no definitions of palliative nursing.

A ‘google’ web search was also conducted for the terms palliative nursing, end of life care self-care, and self-management support. According to Web search workshop a UK consultancy service for optimising and marketing of websites users searching on the internet rarely go beyond the top 30 results [18]. Therefore, only the top 30 results were reviewed for keywords which always took longer than the 30 minutes to review the first 30 sites.

This search string was then combined using Boolean operator ‘OR’ and ‘AND’. Searches were limited only to the English language. Reference lists of all identified papers were scrutinised, hand searches of international journal of palliative nursing, grey literature and key websites was also conducted, using google and google scholar. The University of Dundee Library Catalogue and google scholar were also searched for key textbooks and book chapters. Additional websites were identified via links identified within the Google search and in collaboration with subject experts revealed additional websites, which yielded 11 potentially useful definitions or concepts. The top 30 results of a Google search were reviewed for the keywords of self-care and self-management support revealed 16 relevant articles. Key terms and words included self-care, self-management, self-efficacy and self-help. To place self-management in a professional context the palliative care and palliative nursing literature was examined to further elicit usage of the terms self-care and self-management (Google search Table 3).

**Table 3:** Google search and results

**Google search term**	**Number of useful results**
Self care	8
Self management support	8
Palliative care	8
Terminally ill	3
Terminal care	4
Hospice	11
Life limiting illness	7
Palliative nursing	5
End of life care	7

Inclusion and exclusion was applied to ensure that only relevant publications were included in the review (Table 4). All titles and abstracts returned from the initial search were independently appraised by four authors. Full articles were obtained and appraised if they met the inclusion criteria.

**Table 4:** Inclusion criteria for literature review

**Inclusion**	**Rationale**
Published between 1990-2013	It was necessary to put time limits on the review. It was also determined that the majority of relevant literature was published during this period
English text	Due to budgetary constraints text other than English was excluded
Described the results of empirical research publications	Opinion or theoretical pieces were not included. It was determined that a more comprehensive review would be obtained if only empirical papers were used
Adults only	Palliative care and self care issues affecting children are different and palliative care services for adults and children are different, therefore only studies relating to adults were included

Defining attributes palliative nurse

Supportive

Intensive caring, continuous knowing and continuous giving

Fostering hope

Providing comfort

Proving an empathic relationship

Being there

Acting on the patients behalf

Meeting the patients’ needs

Working together/teamwork

Knows what they are doing

Knowing the patient

Dignity

Providing information

## Results

### Results reviewed by BJ and ER

#### Dictionary definitions

The concept analysis framework identified by Walker and Avant indicates that definitions of terms are first sought as dictionary definitions *as part of identifying the concept.*

### Palliative

Palliate has its origins in medieval Latin *palliativus*, from the verb *palliare* ‘to cloak’.

#### *Adjective*

(of a medicine or medical care) relieving pain without dealing with the cause of the condition: *orthodox medicines tend to be palliative rather than curative.*

(of an action) intended to alleviate a problem without addressing the underlying cause: *short-term palliative measures had been taken.*

#### *Noun*

A palliative medicine, measure, etc.: *antibiotics and other palliatives social projects presented as palliatives for the urban crisis oxford*[19].

### Self-care

Self-care has been defined in the dictionary as: “The care of oneself without medical, professional, or other assistance or oversight” [20].

### Self management support

Self-management can be defined as the decisions and behaviours that patients with chronic illness engage in that affect their health. Self-management support is the care and encouragement provided to people with chronic conditions and their families to help them understand their central role in managing their illness, make informed decisions about care, and engage in healthy behaviours [21].

Self-management is ‘the successful outcome of the person and all appropriate individuals and services working together to support him or her to deal with the very real implications of living the rest of their life with one or more long term condition’ [22].

Support for self management is what services provide to encourage people to take decisions and make choices that improve their health, wellbeing and health-related behaviours [22].

### Literature definitions of palliative care, palliative nursing and self managent support

#### Palliative care

Palliative care is among one of the fastest-growing specializations globally in the fields of nursing and medical education and referred to by current International government documents as not only focussing on death and dying but also on improving the quality of life for the patient and family [23,24]. For almost a generation, to varying degrees, palliative care has been associated with total, active, holistic and therapeutic intervention/s, which focus on the quality of life for the patient and his/her family [24,25]. The universal worldwide accepted definition is that proffered by the World Health Organisation (WHO) [26]. The WHO state that palliative care is an approach that improves the quality of life of patients and their families facing the problem associated with life threatening illness through the prevention and relief of suffering by means of early identification and impeccable assessment and treatment of pain and other problems, physical, psychological and spiritual.

Palliative care:

•provides relief from pain and other distressing symptoms;

•affirms life and regards dying as a normal process;

•intends neither to hasten or postpone death;

•integrates the psychological and spiritual aspects of patient care;

•offers a support system to help patients live as actively as possible until death;

•offers a support system to help the family cope during the patients illness and in their own bereavement;

•uses a team approach to address the needs of patients and their families, including bereavement counselling, if indicated;

•will enhance quality of life, and may also positively influence the course of illness;

•is applicable early in the course of illness, in conjunction with other therapies that are intended to prolong life, such as chemotherapy or radiation therapy, and includes those investigations needed to better understand and manage distressing clinical complications” [26].

### Palliative nursing

The development of palliative care nursing has been part of a movement that has grown from roots in the nineteenth century, and particularly the second half of the twentieth century through the UK hospice movement and principally Cicely Saunders, who was originally a nurse [27] Seymour [28] argues that one of the clearest definitions of palliative nursing is that of Johnston [27] p. 2): “All life-threatening illnesses – be they cancer, neurological, cardiac or respiratory disease – have implications for physical, social, psychological and spiritual health, for both the individual and their family. The role of palliative nursing is therefore to assess needs in each of these areas and to plan, implement and evaluate appropriate interventions. It aims to improve the quality of life and to enable a dignified death” [29].

The palliative nurse, therefore, enters into a unique therapeutic relationship with the patient, which requires excellent communication skills and emphasises role aspects such as educator and information giver [27,29,30] and highlights their key involvement in the delivery of individualised, holistic care [27,30]. The expert palliative nurse is someone who is interpersonally skilled, particularly in terms of the ability to be willing to listen, has personal humane characteristics such as warmth, kindness and compassion, and who helps the patient by meeting their needs, is there for them and provides them with emotional support, knows the patient as person and is knowledgeable, in particular, about pain and symptom control [27].

### Self-management support

As discussed previously, the political and professional agenda over the last decade has changed favourably in terms in of integrating self-management into the health care agenda. This interest has the potential to benefit greatly palliative care delivery and the development of palliative care services. The challenge remains, however, as to how this is accomplished and, in particular, how best self-management can be implemented in practice. It is recognised that incorporating the concept of self-management into palliative nursing practice brings additional challenges when managing symptoms at the end of life and when there is no known cure [7]. However, it is understood that if self-management can be utilised to a greater degree it will result in a better quality of life for patients and their families and may reduce the financial costs [7,28].

Self-management has for a long time been associated with a process whereby patients deliberately act on their own behalf in health promotion and prevention of illness and the detection and treatment of health deviations [28]. It has, however, historically taken second place to the medicalisation of disease and the patient’s passive acceptance of the care given by the medical and nursing professions. In examining the future potential of the concept for palliative care practice two observations are relevant. Firstly, up to the first decade of the twenty-first century most self-management strategies reviewed in the literature were professionally initiated and led [2]. Secondly, from a research perspective, few studies up to 2005 appeared to incorporate the patient’s views on palliative nursing care, particularly the concept of the expert palliative nurse [27].

Nevertheless, the concept of self-management is not unique to nursing practice as the concept was identified in the latter half of the twentieth century as part of the development of nursing theory and models to define and support the principles of nursing practice linked to other concepts such as coping [31,32]. Orem in particular championed the concept of self-management and defined it as the supported activities of individuals, in order to maintain health and wellbeing. Her research identified that deficits in self-management were often related to factors such as lack of knowledge, side effects of treatment, or physical, social and psychological aspects related specific to the individual [33]. These factors remain central to the consideration of self-management in the context of palliative nursing and Orem’s model has relevance to palliative nursing as it links the concept to aspects of self-management in the contemporary literature, particularly in relation to wellbeing. Self-management in general has been shown to improve health outcomes, promote a feeling of well-being and improve the quality of life for those suffering incurable conditions [28].

A useful broad starting point to clarifying the meaning, relevance and use of the concept self-management in the context of palliative care and palliative nursing, is the definition put forward by Corner [34] (p.516). In palliative care the goal is to ‘live with dying’ with the focus on the self and not just the physical effects of illness. the following definition is, therefore, appropriate: maintaining ones usual practices of self-care, those things that are important and unique to oneself in maintaining ones sense of self; being given the means to master or deal with problems, rather than relinquish them to others”. This definition emphasises the patient; ‘being in control’ and ‘maintaining independence’, which are important in end of life care [27]. This definition immediately places the patient at the centre of the care and caring processes. However, it also introduces the idea of self-management as part of the caring process. In countries where the focus of health care has moved from hospital to the community, many patients desire to be cared for at home, whenever possible, and the goal is often concerned with achieving patient and family choice [35]. This provides an ideal opportunity for the individual and his/her family to be involved fully in and have control of, their care. Health care policy has reinforced this objective incorporating self-management as an additional focus with emphasis on enabling patients to *manage* their own health and well-being [6,35,36]. While willingness and ability to self-management will change over time, it is also affected by the unpredictable nature and complexity of health related challenges with the person receiving palliative care [37].

Self-management challenges may be compounded by the plethora of information which is now available in the public domain. This is increasing more recently, with ease of access through information technology. These developments bring with them the potential of patients to access incorrect information [38]. Informed decision-making and knowing the patient’s preferred choice [37] stress the importance of open and collaborative dialogue and knowledge of the patient’s own story past and present. These recommendations imply that palliative care is a continuous process enabling the patient to cope with and respond appropriately to challenges as and when they arise and make choices and decisions about the future. These key aspects of self-management highlight the importance of ‘knowing’ the patient as a person [27]. They also highlight that in the field of palliative care, the facilitation of self-management brings additional challenges in managing symptoms and helping patients to live a life focused on quality as opposed to quantity (time).

Moreover, an important issue for using self-management in practice is that not all individuals are either able to, or wish to, engage fully in self-management activities and that part of the professional’s assessment is to identify the degree of self-management need and capability that is appropriate at any point in time [2]. Degrees of self-management engagement can be identified through robust physical and emotional management that enables the individual to adjust and match their self-management capability to their identified self-management needs, thus enabling them to stay in control of their unique and individual situation. Table 5 identifies the essential themes aimed at initiating and supporting self-management actions [8].

**Table 5:** **Supporting self-management: themes and sub themes**[8]

**Theme**	**Sub-theme**
Maintaining normality	Goal setting; How others treat you; Maintain normality-taking a break/holiday
Preparing for death	Euthanasia; Getting worse; Leaving family behind; Planning funeral; Process of dying
Support from family/friends	Carer support/information; Talking about difficult issues; Respite
Self-cares strategies/physical	Activities of daily living management; Aids to house; Complementary therapy; Financial help benefits; Managing symptoms
Self-care strategies/emotional	Accepting; Being positive; Choice; Control; Religion; Support from others with cancer
Support from health professionals	Clinical nurse specialist fixer/coordinator; Home help carer; Hospice day care; Out-of-hours care

Various attempts have been made to clarify the terms self-management. For instance, it has been viewed as a transition and how people incorporate the consequences of illness into their lives [39]. As well as, associating the concept with the professional support and direction patients receive, including how to follow given instructions and manage other aspects of their condition [28,31]. While these definitions allow to patients a semi-passive role in their care; they are also associated with active patient and professional collaboration in decision making, facilitating choice and decisions that support independent patient activities. While many of these factors are relevant to the palliative care context Johnston et al. [2] identify the description by Foster et al. [39] as most appropriate to palliative care as it highlights strategies used by individuals to enhance control and maximize wellbeing and the effects and approaches used by the individual to optimise living as closer than any other to being appropriate for people with advanced disease at the end of life.

The capability to manage self-management appears to be associated strongly with the use of effective and robust assessment techniques, tools and processes, which are dependent on the patient with support from the nurse, assessing their own self-management needs and capability [2,8,28]. In addition, self-management in a palliative care context is linked to capability to control pain, manage other symptoms as well as evaluating the effectiveness of interventions.

The concept of ‘supported self-management’ can, therefore, be said to embrace both self-care and self-management. In ‘supported self-management’ the concept of self-management can be linked closely to the patient’s capability, while the professional is facilitating the patient to assess and identify their needs, moreover, self-management can be linked to outcomes of care and the patient’s actual and potential capability to act in a way that meets their identified needs.

Palliative care, therefore, needs to be underpinned by robust needs assessment, by the nurse, considering the patient’s wishes, skills, behaviours and knowledge. The fundamental concepts underpinning palliative nursing assessment are that it is, *“… dynamic, Individualised, patient and family centred, sensitive and appropriate, holistic, therapeutic, contextual, comprehensive, based on reliable, current and valid information, evidence-based, driven by and focussed on process and outcomes”*[27]. This definition of assessment can be used to guide the professional to achieve the goal of supported self-management as a contemporary concept, with strong underpinnings in the effectiveness of the patient/professional relationship and the skills of the professional to support the patient in his/her self-management endeavours [5].

Assessment also brings into sharp focus the effectiveness of the Multi-Disciplinary Team (MDT) in supporting self-management. Johnston [8,27] highlights the importance of effective collaboration and communication in supported self-management, which could be considered as the heart of the Multi-Disciplinary Team’s (MDT).

Key characteristics of self-management support in palliative nursing are, therefore, presented in Table 6 according to the Walker and Avant theoretical process.

**Table 6:** Attributes of self-management and nursing role with author

**Attribute**	**Nursing role**	**Author**
Maintaining normality	Knowing the patient	Jarret et al., Skilbeck et al.
Preparing for death	Support	Johnston et al., O’Berle and Davies
	Being there	Zabalgui
	Comfort	
	Excellent communication skills	Johnston et al.
		O’Berle and Davies
Support from family/friends	Emotional support	Zabalegui
Self-care strategies/physical	Promoting independence	Johnston et al., Rhodes et al.
	Good pain and symptom control	Rhodes et al.
Self-care strategies/emotional	Promoting independence	Johnston et al., Rhodes et al.
	support	
Support from health professionals	Teamwork	Johnston et al.
	Referral role	
	Collaborating providing information	Skilbeck et al.

### Antecedents and consequences

The identification of antecedents and consequences helps to refine the critical attributes and elucidate the contexts in which the concept is generally used [16]. Antecedents are factors that must be present before the occurrence of the concept whereas consequences are events that occur as a result of the concept.

For supportive self-management in palliative nursing to occur we identified four antecedents; *presence of the nurse* and *spending time with the patient*; [27,40]*development of a relationship with the patient,*[8,27,40-44]. *Skills, knowledge and expertise of the nurse*; [45-49], *team working and the ability of nurse to recognise when to refer on to other professionals or support services*.

In analysing the concept of supportive self-management and palliative nursing this requires the demonstration of events that occur as a result of support being experienced/delivered. These consequences can be either a positive or negative experience for patients. Positive experiences for patients include feeling cared for and having their needs met; [27,46] being informed [27,47-50] and being supported [27,48]. Negative experiences include pain and symptom control needs not met [51,52] not having their needs met and not being supported, [27,42,43] as wellbeing unable to manage, particular in relation to activities of daily living [10].

## Discussion

### Strengths and limitations of the method

The purpose of this analysis was to define the concept of self management support in relation to palliative nursing. Whist literature was retrieved from a variety of sources, certain limitations are worth mentioning. Literature in languages other than English were not accessed. A broader search could have produced a more comprehensive definition. Additionally, an alternative concept analysis method may have produced a different outcome.

This concept analysis has identified that there is no universal definition of supportive self-management in palliative nursing. A clarified definition for nursing use based on this concept analysis is proposed below.

Supportive self-management in palliative nursing is; *assessing, planning, and implementing appropriate care to enable the patient to live until they die and supporting the patient to be given the means to master or deal with their illness or their effects of their illness themselves.*

The use of a model such as that proposed by Walker and Avant [16] was found to be beneficial in facilitating a systematic approach to literature retrieval, review and analysis. While stages 1 and 2 of the model were used to clarify the direction of travel and inform key words and terms for the literature review stages 3 – 5 and 7 informed the focus of the analysis as well as the breadth and depth of literature retrieved. Stage 8 was important to supporting synthesis, which re-define self-management. Stages eight of the model helped to focus attention on the relevance and applicability of self-management to practice.

## Conclusions

**Figure 1:**
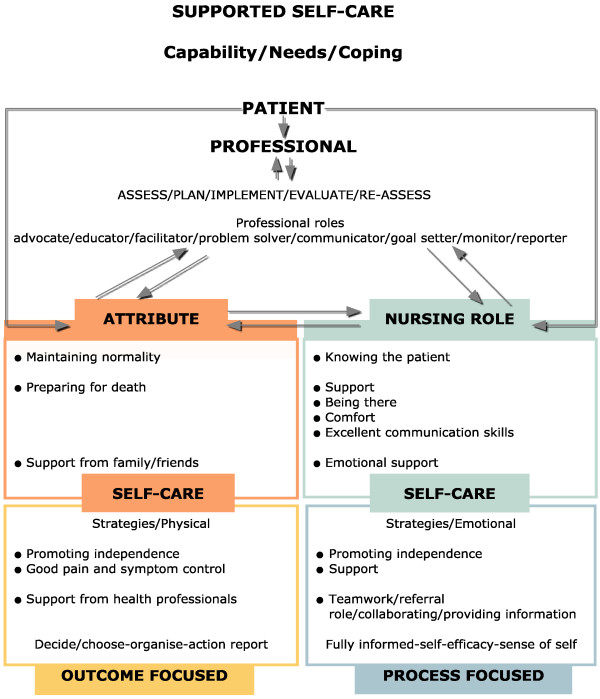
Characteristics and attributes of palliative nursing and supported self care.

For this concept analysis references to self-management were drawn mainly from chronic and disease management literature, as well as, the few articles on self-management available in the palliative care field. Self-management has been conceptualised in relation to how patients can gain and remain in control of their care process and how professionals can support this process. The concept analysis led to consideration of the extended concept of ‘supported self-management’. The important role nurses have in supporting patients with palliative care needs to be able to maintain normality and independence and be in control for as long as possible is highlighted and to be encouraged. Figure 1 is proposed as a guide to aid current discussion, for clinical practice and to aid further research and development of the concept ‘supported self-management’ in palliative care nursing and education.

## Competing interests

The authors declare that they have no competing interests.

## Authors’ contributions

BJ devised the concept analysis and wrote the first draft ER contributed to the first draft and edited future drafts AB and JM and PC helped devise the concept analysis and edited the first draft. All authors read and approved the final manuscript.

## Pre-publication history

The pre-publication history for this paper can be accessed here:

http://www.biomedcentral.com/1472-6955/13/21/prepub
